# A geo-gender-based analysis of human health: The presence of cut flower farms can attenuate pesticide exposure in African communities, with women being the most vulnerable

**DOI:** 10.7189/jogh.14.04064

**Published:** 2024-10-11

**Authors:** Irena F Creed, Kevin J Erratt, Phaedra Henley, Pamela F Tsimbiri, John R Bend, William A. Shivoga, Charles G Trick

**Affiliations:** 1 Department of Physical & Environmental Sciences, University of Toronto, Toronto, Ontario, Canada; 2 Center for One Health, University of Global Health Equity, Butaro, Rwanda; 3 Department of Reproductive Health, Faculty of Health Sciences, Egerton University, Egerton, Kenya; 4 Department of Pathology and Laboratory Medicine, Schulich School of Medicine and Dentistry, Western University, Ontario, Canada; 5 Department of Biological Sciences, Centre of Excellence for Water and Environment Resources Management (CEWERM), Kakamega, Kenya; 6 Department of Health & Society, University of Toronto, Toronto, Ontario, Canada

## Abstract

**Background:**

The rapid expansion of the cut flower industry in Africa has led to pervasive use and potential exposure of pesticides, raising concerns for local communities. Whether the risks associated with pesticide applications are localised or have broader implications remains unclear.

**Methods:**

We measured biomarkers of real and perceived pesticide exposure in two Kenyan communities: Naivasha, where the cut flower industry is present, and Mogotio, where the cut flower industry is absent. We measured real exposure by the percentage of acetylcholinesterase (AChE) inhibition and perceived exposure by assessing hair cortisol levels, a biomarker of stress. Additionally, we conducted a demographic survey to evaluate the health and socioeconomic status of participants, as well as their perceptions of pesticide risks associated with the cut flower industry.

**Results:**

Perceived pesticide exposure was more common in Naivasha (n = 36, 56%) compared to Mogotio (n = 0, 0%), according to community surveys. However, Mogotio residents had significantly higher mean hair cortisol levels (mean (x̄) = 790 ng/g, standard deviation (SD) = 233) and percentage of AChE inhibition (x̄ = 28.5%, SD = 7.3) compared to Naivasha residents, who had lower mean hair cortisol levels (x̄ = 548 ng/g, SD = 187) and percentage of AChE inhibition (x̄ = 14.5%, SD = 10.1). Location (proximity to cut flower farms) and gender were significant factors influencing pesticide exposure, with individuals living outside the cut flower industrial complexes being at higher risk. Women in both communities were the most vulnerable demographic, showing significantly higher mean hair cortisol levels (x̄ = 646 ng/g, SD = 267.4) and percentage of AChE inhibition (x̄ = 22.5%, SD = 12.4) compared to men hair cortisol levels (x̄ = 558.2 ng/g, SD = 208.2) and percentage of AChE inhibition (x̄ = 10.4%, SD = 13.1).

**Conclusions:**

A heightened awareness of the potential risks of pesticide exposure was widespread within cut flower industrial complexes. This may have led to a reduction in exposure of both workers and non-workers living within or close to these complexes. In contrast, communities living outside these complexes showed higher levels of exposure, possibly due to limited chemical awareness and a lack of precautionary measures. Despite this contrast between communities, women remained the most vulnerable members, likely due to their socioeconomic roles in African society. Monitoring women's pesticide exposure is crucial for providing an early warning system for community exposure.

Cut flower farms are widespread in sub-Saharan Africa, generating considerable export revenue and employing hundreds of thousands of people (**Figure 1**). One consequence of the increased cut flower production is the concurrent increase in pesticide applications [1,2]. Pesticide use results in the possible exposure of workers to toxic chemicals along the supply chain. However, there is also the increased availability of pesticides in the surrounding communities [3]. Organophosphate (OP) and carbamate pesticides are commonly used in the African cut flower industry and can serve as an ecotoxicological tracer for exposure [4,5]. They inhibit acetylcholinesterase (AChE), an enzyme that breaks down the neurotransmitter acetylcholine to terminate synaptic transmission [6]. When AChE is inhibited, there is a build-up of acetylcholine, causing interminable muscle contractions and neuromuscular paralysis that can eventually lead to death by respiratory distress and cessation in highly exposed individuals [5,7]. The effects of chronic exposure to OPs and carbamates have not been fully elucidated, but have been linked to increased incidences of upper and lower respiratory symptoms, suicidal ideation, neuropsychiatric disorders, sensory ataxia, and other delayed neuropathies [5,7,8]. The use of these pesticides on flower farms has caught international attention in the media, with concerned consumers demanding a safer product for African flower farm workers [9].

**Figure 1:**
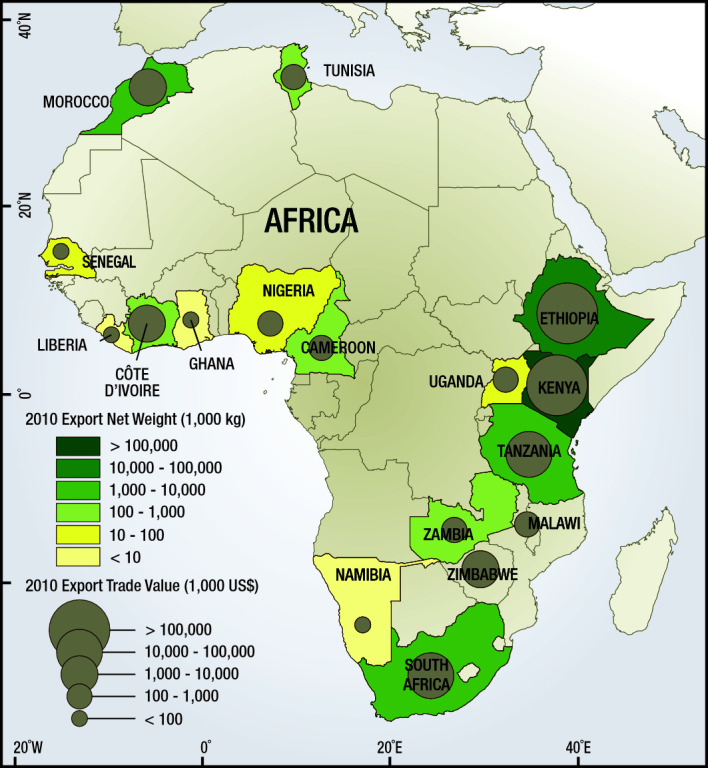
Export trade values (USD) and export net weight (kg) of cut flowers reported to the United Nations commodity trade database in 2010 for countries in Africa.

Adverse human health effects from real exposure to agricultural pesticides exist; however, there are also negative human health impacts from the perceived risk of being exposed to chemicals, which contribute to overall psychosocial stress. Chemophobia, defined as the ‘irrational fear of chemicals’, can be rooted in either unfounded or founded concerns, where each can lead to psychosocial stress and stress-associated illnesses [10,11]. It has become widespread in modern society, despite the inherent risks of chemical exposure in the workforce dropping sharply in response to the widespread adoption of workplace interventions to reduce exposure [11].

There is a need for biomarkers that differentiate between real and perceived exposure to pesticides. For real exposure, inhibition of red blood cell (RBC) AChE reflects the biological effects of OPs on the central nervous system in a dose-dependent manner [5]. Small, but reliable decreases in RBC AChE activity in populations chronically exposed to OPs have been previously reported [8,12]. Typically, RBC AChE inhibition is measured in two steps: first, a measurement is taken before potential pesticide exposure, followed by a second measurement afterward. However, inhibition of RBC AChE can also be assessed by comparing it to a standard value [7]. For example, if a person’s RBC AChE level falls below 20% of the standard value, it indicates likely exposure to an anticholinesterase [13]. RBC AChE levels can be used to evaluate chronic exposure to OPs as its inhibition can last several months after exposure [4,7]. In contrast, cortisol concentrations in hair can be used as an indicator of perceived stress related to chemical exposure, and are increasingly used worldwide as a biomarker of chronic psychosocial stress, including in sub-Saharan African communities [14,15].

In this study, we used a community-based participatory approach [16] to explore the real vs. perceived risks of pesticide exposure to cut flower farm workers and the communities surrounding the cut flower industry in Lake Naivasha, Kenya (**Figure 2**). The cut flower industry in Lake Naivasha contributed up to 70% of Kenya's floriculture exports in 2010 [17,18]. Originating in the 1980s with a local population of 44 000, the establishment of the cut flower industry led to a significant population growth, reaching approximately 380 000 by 2010 – an increase of about 750%. The cut flower industry had expanded to over 4000 hectares and employed about 100 000 people in 2010, constituting roughly a quarter of the population [19,20].

**Figure 2:**
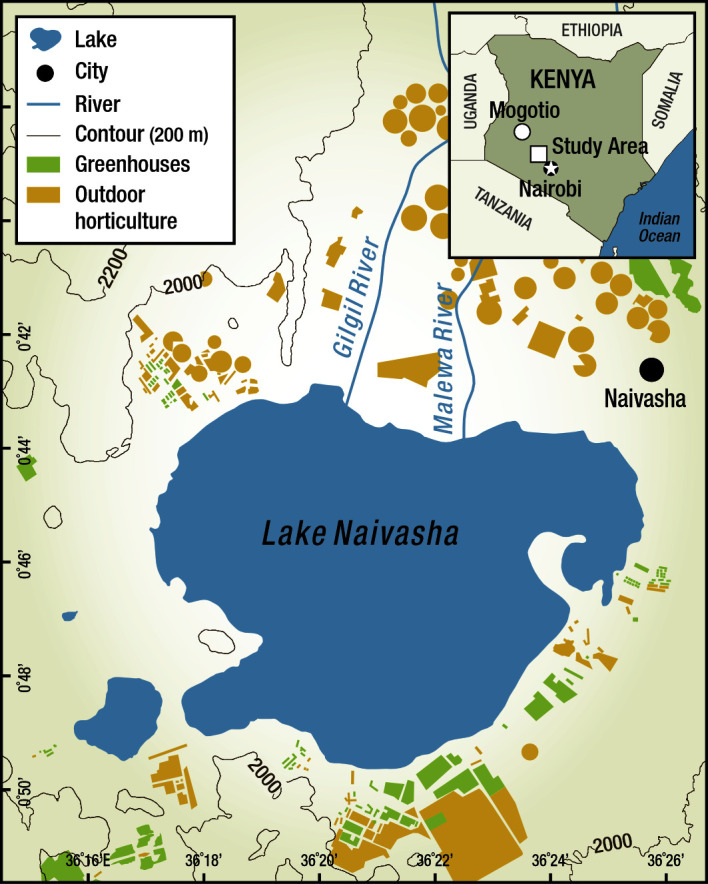
Map of Naivasha, Kenya.

In this study, we tested the hypothesis that the cut flower industry in Naivasha is a major source of both real and perceived risks of pesticide exposure. We also explored whether the relative contributions of real and perceived risks from pesticide exposure differ between contrasting communities: Naivasha, located within the cut flower industrial complex, and Mogotio, which lies outside the industrial zone. The key question we addressed is which population subset is the most susceptible to pesticide exposure. Furthermore, we examined the socioeconomic factors that may attenuate the negative health effects of such exposure.

## METHODS

### Study design

We conducted a case-control design comparing pesticide exposure between communities located within (Naivasha) and outside (Mogotio) a cut flower industrial complex. Naivasha hosts one-third of the cut flower farm operations in Kenya, informally referred to as the ‘flower basket of Kenya’ [1]. It had a population of 380 000, with a population density (i.e. people per square kilometre of land area) of 182/km^2^ in 2010 [21]. Naivasha has an agriculture-based economy, with floriculture and horticulture as primary activities [19]. We considered the presence of a cut flower industrial complex and residence dependency on this industry for employment as factors that may increase the risk of pesticide exposure. Mogotio has a population of 91 100, with a population density of 66/km^2^ in 2010 [21]. It is an agricultural-based economy, with livestock rearing and dairy farming as the main activities, as well as maize and sisal cultivation [22]. We chose Mogotio as the control population because its socioeconomic and environmental conditions are similar to Naivasha, and because of its geographic distance (110 km) from the cut flower industry. We considered the lack of a cut flower industry and the lack of occupation within this industry as factors that may contribute to lower risks of pesticide exposure.

### Survey instrument

We assessed health and socioeconomic status of Naivasha and Mogotio residents by administering an identical survey for both populations. The development and implementation of said survey were described previously [1]. The survey tool was assessed for clarity and quality through several focus group discussions, and was also pilot-tested (n = 20) in Kaptembwa, Nakuru, Kenya, with no issues discovered. Locally trained clinicians administered each survey verbally in Kiswahili or English to 800 participants in Naivasha and 200 participants in Mogotio. The surveys gathered information on demographics (e.g. age, gender, marital status), occupation (e.g. cut flower farmer employment), education, living conditions (e.g. number of residences, homeowner status (rented/owned)), and perceived pesticide exposure.

### Biomarkers

Of the 1000 survey respondents in a previous study [15], 200 were randomly selected, with each participant provided with an identification code. Of the 200 participants, 89 provided hair and blood samples. The number of samples was <200, as some individuals preferred not to or could not give blood or hair samples (e.g. shaved heads). Hair cortisol concentrations were used as a biomarker of psychosocial stress (i.e. perceived exposure biomarker), as they are an established biomarker to assess cumulative stress exposure over the short term (3–6 months). This non-invasive method is advantageous as hair cortisol remains highly stable for years at ambient temperature, facilitating long-term storage [14]. The percentage of AChE inhibition in RBC was used as a biomarker of pesticide exposure (i.e. real exposure biomarker). Notably, OP and carbamate pesticides are recognised for their ability to inhibit AChE and are the primary agents used in the African cut flower industry. The percentage of AChE inhibition functions as a useful ecotoxicological marker for pesticide exposure, due to the high sensitivity of AChE to pesticides, even at trace levels, and its dose-dependent response characteristics [6].

### Perceived exposure biomarker

We collected hair from the posterior vertex region as close to the scalp as possible. We wore gloves and used scissors which we cleaned and disinfected with isopropyl alcohol and dried between each use. After collection, we inserted the hair sample into an envelope, ensuring that it was not bent, and sealed the envelope with tape. Hair samples were collected from 89 participants – 64 from Naivasha and 25 from Mogotio.

The procedure for analysing hair for cortisol content has been detailed previously [15]. We weighed the hair with an analytical balance and transferred a 10–15 mg portion into a glass scintillation vial. We washed each sample three times with HPLC-grade isopropanol and left it to dry for at least five hours. Afterwards, we added 1 mL of HPLC-grade methanol and finely minced hair using surgical scissors. We then sealed the vials and incubated them in a rotating incubator at 50°C and 200 RPM for 16 hours. The vials were then cooled to room temperature. We transferred the methanol extract to a glass tube, and the solvent evaporated from each sample by heating it to 50°C under a gentle stream of nitrogen gas. We reconstituted the remaining residue by adding 250 μL phosphate-buffered saline solution to the sample and vortexed it until well mixed. Subsequently, we added 50 μL of the buffer solution to the wells in duplicate, following the instructions provided in the enzyme-linked immunosorbent assay (ELISA) kit (ALPCO Diagnostics, NH, US). We conducted the ELISA assay on a flat-bottomed, antibody-coated 96-well plate, and read the absorption on a Vmax plate reader (Molecular Devices, CA, US) at 450 nm. Hair cortisol content was provided in ng of cortisol/g of hair (ng/g).

### Real exposure biomarker

Concurrent with hair collection, a certified clinician collected whole blood in BD Vacutainers containing 10.8 mg K2EDTA to measure levels of RBC AChE with the World Health Organization (WHO)-approved AChE kit (EQM Research Test-mate AChE Cholinesterase Test System Model 400, Cincinnati, OH, US). The blood collection area was thoroughly disinfected to prevent contamination. All participants washed their hands before sample collection to prevent contamination from other sources, and their arm was disinfected at the point of puncture before blood collection. In total, we collected and analysed 89 blood samples – 64 from Naivasha and 25 from Mogotio.

We measured RBC AChE activity within minutes of collection using a 10 μL capillary of anti-coagulated fresh whole blood. The WHO-approved field kit is intended to assess asymptomatic pesticide poisoning and consists of a battery-operated portable AChE testing system that uses the modified Ellman method for measuring cholinesterase activity (Ballantyne and Marrs, 2017). The amount of yellow colour produced as a result of the Ellman reaction is measured with a spectrophotometer, indicating the presence of cholinesterase activity. The Test-mate kit provides a standardised normal AChE (U/mL) value of 4.71%, which is representative of an unaffected individual. AChE inhibition in RBC was calculated by dividing the participant's AChE (U/mL) value by the standardised value, which was multiplied by 100 to obtain the percentage of AChE inhibition ((participant AChE / standard AChE) × 100).

### Statistical analysis

**Figure 3:**
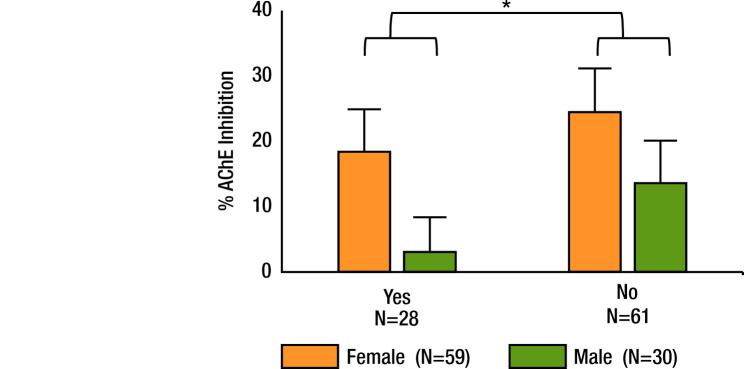
Means and standard deviations of % AChE inhibition in RBC by gender and perceived pesticide exposure. AChE – acetylcholinesterase, RBC – red blood cells.

We determined the statistical significance of differences in perceived pesticide exposure, hair cortisol concentrations, and % RBC AChE inhibition among workers and non-workers of cut flower farms, and among groups from Naivasha and Mogotio. We evaluated the statistical significance (*P* < 0.05) of differences between groups using Student’s *t* tests (**Table 1**). Before performing Student’s *t* tests, we tested the normality of the data with the Shapiro-Wilk normality test, with *P*-values greater than 0.05 indicating a normal distribution. Further, a two-way ANOVA using gender and perceived pesticide exposure as the independent variables was conducted to assess the significance (*P* < 0.05) of these variables on % AChE inhibition (**Figure 3**). The Shapiro-Wilk normality test, student’s *t* tests, and two-way ANOVA were performed using SigmaPlot (Systat Software, San Jose, CA).

**Table 1:** Differences in perceived pesticide exposure, hair cortisol concentrations, and AChE inhibition in RBC by community and occupation

	Perceived pesticide exposure		Hair cortisol concentrations (ng/g)		% RBC AChE inhibition	
	**n (%)**	***P*-value**	**x̄ (SD)**	***P*-value**	**x̄ (SD)**	***P*-value**
**Community**		<0.001		0.029		0.018
Naivasha (n = 64)	36 (56)		548 (187)		14.5 (10.1)	
Mogotio (n = 25)	0 (0)		790 (233)		28.5 (7.3)	
**Occupation**		0.566		0.900		0.117
Flower farm worker (n = 42)	23 (55)		542 (181)		15.6 (11.4)	
Non-flower farm worker (n = 22)	13 (58)		559 (204)		15.7 (15.6)	

AChE – acetylcholinesterase, RBC – red blood cells, SD – standard deviation, x̄ – mean

**Figure 4:**
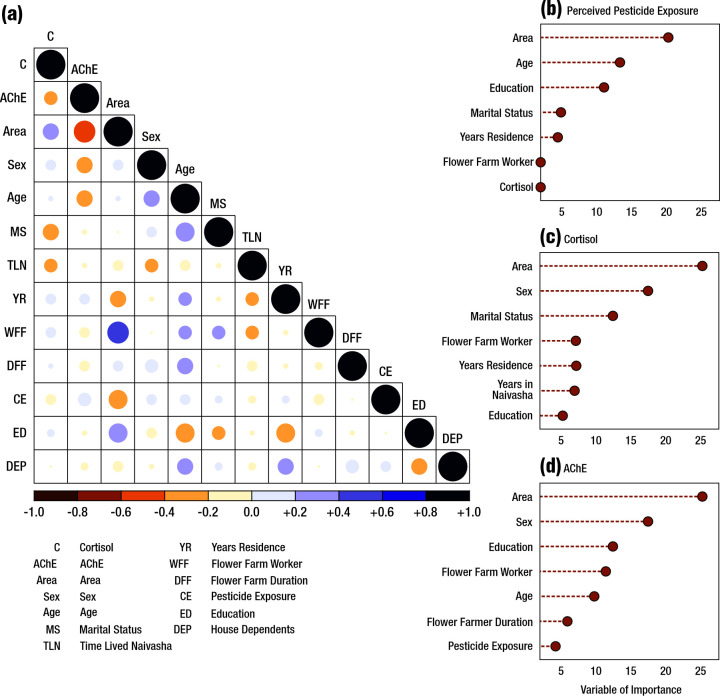
Spearman correlation matrix and random forest models of factors regulating perceived pesticide exposure, cortisol, and AChE. **Panel A.** Spearman correlation matrix of the study variables. **Panel B.** Random forest model of factors regulating perceived pesticide exposure. **Panel C.** Random forest model of factors regulating cortisol. **Panel D.** Random forest model of factors regulating AChE. AChE – acetylcholinesterase.

We used random forest analysis to identify predictor variables of greatest importance for perceived and real pesticide exposure. Before the random forest analysis, we used Spearman rank correlation to detect strongly correlated variables. Multi-collinearity was not detected at a threshold of ±0.6, and no variables were excluded from the analysis. We presented the results of the random forest analysis on a variable importance plot, which displays a hierarchy of variables ranked by their importance, with the most important variables shown at the top (i.e. the top-ranked variable yields the most significant impact on the tested response). Higher %IncMSE equals greater variable importance, with lower %IncMSE variables considered ‘unimportant’ (**Figure 4**). Significance was determined using a %IncMSE score of ≥10, as assigned by the random forest analysis.

Regression trees are a nonparametric analysis [23] that identifies and classifies several independent variables (i.e. the socio-ecological indicators from the survey) involved in the classification of a dependent variable (i.e. hair cortisol content or % AChE inhibition). They produce binary splits based on the Gini index, which serves as a measure of impurity at each node. The independent variables used in the regression tree are those where the split maximises the predictability of the dependent variable at a specific level of the regression tree. The regression trees start with a root node that contains the entire sample of participants. It is then split into several parent and child nodes and, finally, a terminal node when segmentation is no longer possible. Random forest and regression tree analyses were performed using R, version 4.2.1, (R Core Team, Vienna, Austria). We put the predictor variables with %IncMSE>10 into a regression tree analysis to classify the profiles of participants regarding perceived pesticide exposure, hair cortisol content, and % AChE inhibition.

### Ethical approval

The Kenya Medical Research Institute and the Research Ethics Board of Western University approved the research protocol.

## RESULTS

### Survey instrument

**Figure 5:**
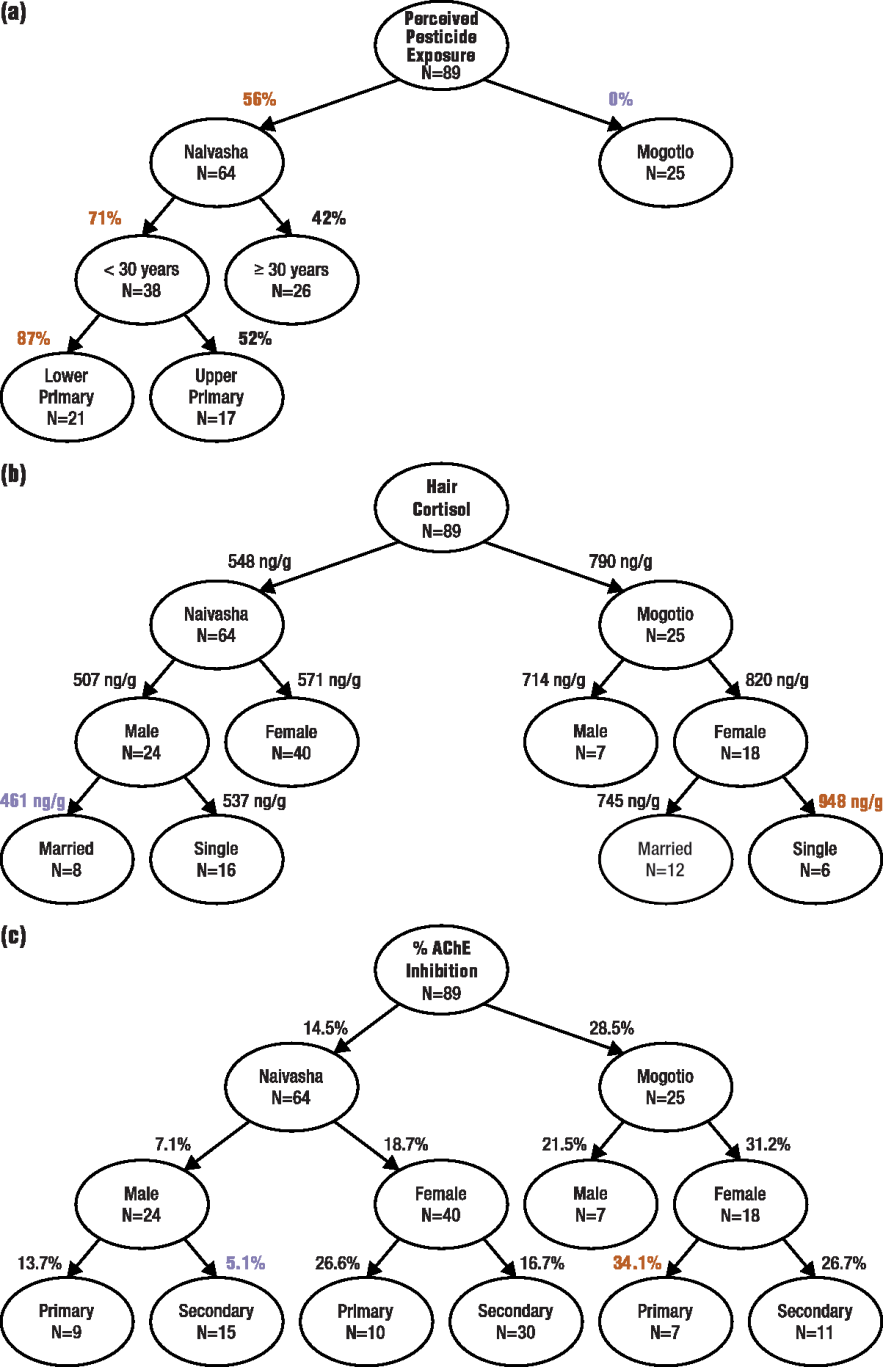
Regression trees of perceived pesticide exposure, hair cortisol levels, and perceived AChE inhibition in RBC. **Panel A.** Regression tree of perceived pesticide exposure (‘yes’ vs ‘no’). **Panel B**. Regression tree of hair cortisol levels (ng/g). **Panel C.** Regression tree of % AChE inhibition in RBC. AChE – acetylcholinesterase, RBC – red blood cells.

The response to the question from our survey ‘Do you think you are being affected by pesticides?’ was used as the variable representing perceived pesticide exposure. The primary socio-ecological indicator of perceived pesticide exposure was found to be geographic location; significantly more people from Naivasha (n = 36, 56%) responded ‘yes’ to the given question, compared to those from Mogotio (n = 0, 0%) (**Table 1**, **Figure 5****,** Panel A). In Naivasha, there was no significant difference in perceived pesticide exposure between those working on the cut flower farms compared to those who did not work on the cut flower farms; respondents representing the entire Lake Naivasha basin felt at risk from pesticide exposure (**Table 1**). Respondents who believed they were being affected by pesticides were typically younger (i.e. age <30 years) and at a lower education status (i.e. lower primary) (**Figure 5**, Panel A).

### Biomarkers of perceived vs real exposure to pesticides

Mean hair cortisol concentrations were significantly different between respondents from Naivasha (mean (x̄) = 548 ng/g, standard deviation (SD) = 187) and Mogotio (x̄ = 790 ng/g, SD = 233). No significant differences were observed between those respondents from Naivasha who worked on the cut flower farms (x̄ = 542 ng/g, SD = 181) and those who did not (x̄ = 559 ng/g, SD = 204) (**Table 1**). Gender and marital status were also important variables influencing psychosocial stress. The highest hair cortisol concentrations were observed in female respondents, with single females who lived outside the cut flower industrial complex showing the highest psychosocial stress (x̄ = 948 ng/g, SD = 206). In contrast, lower hair cortisol concentrations were observed in male respondents in both locations, with married men living within the cut flower industrial complex showing the lowest psychosocial stress (x̄ = 478 ng/g, SD = 153) (**Figure 5**, Panel B).

Respondents from Naivasha had lower exposure to anticholinesterase pesticides than respondents from Mogotio. There were no significant differences between the % RBC AChE inhibition between Naivasha respondents who worked on the cut flower farms (x̄ = 15.6%, SD = 11.4) and those who did not work on the cut flower farms (x̄ = 15.7%, SD = 15.6) at Naivasha (**Table 1**). Further, % AChE inhibition in RBC was significantly less in respondents from Naivasha (x̄ = 14.5%, SD = 10.1) than in Mogotio (x̄ = 28.5%, SD = 7.3) (**Table 1**). Gender played a key role in understanding pesticide exposure, with women in both regions having higher AChE inhibition than men (**Figure 5**, Panel C). Education also played a key role, with lower education contributing to lower AChE content in both females and males (**Figure 5**, Panel C). Respondents with the highest AChE inhibition were female Mogotio residents who were at a lower educational status (i.e. lower primary) (x̄ = 34.1%, SD = 8.5) (**Figure 5**, Panel C). In contrast, respondents with the lowest AChE inhibition were male, with men with higher education who lived within the cut flower industrial complex having the lowest AChE inhibition (x̄ = 5.1%, SD = 6.2) (**Figure 5**, Panel C).

### Chemical awareness becomes a deterrent to exposure

There was significantly higher AChE inhibition in participants who responded ‘no’ to the question ‘Do you think you are being affected by pesticides?’ compared to those who responded ‘yes’ (*P* = 0.007). Female participants demonstrated significantly higher AChE inhibition than male participants (*P* < 0.001). However, there was no significant interaction between gender and perceived pesticide exposure (*P* = 0.458) (**Figure 3**). A key determinant of perceived pesticide exposure in respondents from Naivasha was age and education level, with participants under 30 years of age and with lower completed education (up to lower primary) expressing higher perceived risks to pesticides (**Figure 5**, Panel B).

## DISCUSSION

The risk of pesticide exposure in Naivasha is a global concern, with individuals making explicit accusations of blaming the booming cut flower industry as the source of environmental and health problems [15,24,25]. Cut flower farms in Kenya were established in the 1980s and grew rapidly [19]. Subsequently, in the mid-1990s, studies assessed pesticide exposure in four communities in Kenya, including one in Naivasha [12,26,27]. Naivasha had the greatest inhibition of RBC AChE (36%), indicating elevated pesticide exposure [26]. The legacy of this pesticide exposure for workers in the cut flower industry is that people who live in Naivasha, the hub of Kenya’s cut flower industry, believe that their health is adversely affected by pesticides (**Table 1**).

Both real and perceived pesticide exposure were pervasive and not restricted to Naivasha. Respondents with the highest mean psychosocial stress (i.e. x̄ = 790, SD = 233 ng/g hair cortisol) and exposure to pesticides (i.e. x̄ = 28, SD = 7.3% RBC AChE inhibition) were individuals who lived in Mogotio, located outside of the cut flower industrial complex (**Table 1**, **Figure 5**, Panels B–C). In contrast, respondents with lower mean psychosocial stress (i.e. x̄ = 548, SD = 187 ng/g hair cortisol) and pesticide exposure (x̄ = 14.5, SD = 10.1% RBC AChE inhibition) were those who lived in Naivasha, located within the cut flower industrial complex (**Figure 5**, Panels B–C). Those who had perceived pesticide exposure had lower real exposure, with education as an important factor for perceived pesticide exposure (i.e. reducing risks). Lastly, women in both regions displayed higher real and perceived pesticide exposure.

### Chemical awareness limits pesticide exposure

We tested the hypothesis that the cut flower industry was the most important source of pesticide exposure. Our findings do not support this hypothesis. Exposure in the highest pesticide application and use area was likely neutralised by occupational training and chemical awareness that extends throughout the community. Survey respondents who worked on the cut flower farms had among the lowest pesticide exposure (i.e. lowest % AChE inhibition), reflecting changes in the pattern of pesticide use at the Naivasha cut flower farms since the mid-1990s [9]. In contrast to previous studies [12], our results showed a reduction exceeding 50% AChE inhibition in Naivasha. We observed a decline from 36% AChE inhibition in the 1990s [12] to 14.5% in 2010. This decline may indicate the widespread implementation of pesticide legislation in the early 1990s to address growing health concerns.

Control of the purchase, use, and regulation of pesticides in Kenya has advanced significantly since the 1990s [9]. This fact, as well as dramatic decreases in the amount of pesticide residue allowed on imports to the EU [28], has forced cut flower farms to follow best practices in pesticide use and release into the environment or face economic repercussions [9]. These changes in pesticide regulation in Kenya and the EU, coupled with the introduction of best-management practices for pesticide application within the Kenyan cut flower industry [29], likely explain why we found no relationship between cut flower industry employment and % AChE inhibition in RBC. It also likely explains why we found a significantly higher % AChE inhibition in Mogotio compared to Naivasha. The % AChE inhibition among Mogotio residents (x̄ = 28, SD = 7.3% RBC AChE inhibition) aligns with values documented in other Kenyan agricultural communities, falling within the range of 26–36% [12]. This alignment may indicate the possibility that best management practices for pesticide use have not been widely adopted in Mogotio.

Survey respondents who worked on the cut flower farms believed they were at risk of pesticide exposure. However, the perception of the risk from pesticide exposure was not related to hair cortisol concentrations. This is likely due to the presence of other stressors that have a more significant impact on chronic stress in this region of the world [15]. The perception of risk of pesticide exposure was related to a smaller % RBC AChE inhibition and thus less pesticide exposure. Specifically, we found significantly lower % RBC AChE inhibition in respondents who responded ‘yes’ to the question ‘Do you think you are being affected by pesticides?’ compared to those who responded ‘no’, suggesting that awareness of pesticide risks is leading to lower pesticide exposure (**Figure 3**). As such, chemical awareness contributes to risk avoidance, resulting in behavioural changes among participants that encouraged precautionary strategies to limit chemical exposure. We found that chemical awareness spreads from cut flower industries, resulting in positive behavioural changes among community members.

Survey respondents with the highest psychosocial stress (i.e. highest hair cortisol concentrations) and highest pesticide exposure (i.e. highest % AChE inhibition in RBC) were those who did not work on the cut flower farms and lived in Mogotio, a settlement removed from any cut flower farms by >100 km. Mogotio residents exhibited a higher % of RBC AChE inhibition than respondents from Naivasha (*P* = 0.016), indicating greater exposure to household or agricultural OP or carbamate pesticides in the community (**Table 1**). These findings suggest a shift from regulated point sources of pesticides (i.e. cut flower farms and radiating out to the surrounding community in Naivasha) to more unregulated diffuse sources of pesticide exposure.

### Women as sentinels

There are many other sources of exposure to OP and carbamate pesticides than the cut flower industry. The application of pesticides in the home (e.g. to eliminate insect pests, including malaria-carrying mosquitoes), in the yard, and on subsistence farms are potential sources of exposure [30]. While strict pesticide regulations exist within the cut flower industry, with employees being trained to minimise the risk of exposure [9], this does not translate to communities where members are often not trained on proper handling and use. Our findings point to elevated risks of non-occupational pesticides among Kenyan residents.

Women are at increased risk of both psychosocial stress and pesticide exposure compared to men, including those who live in, near, and outside the cut flower industrial complex (**Figure 5**, Panels B–C). The increased hair cortisol levels in women may also reflect the reality of being a woman in a historically patriarchal society, where women are often responsible for both reproductive and productive roles, including formal work outside the home, particularly in Naivasha and Mogotio [31]. Marital status emerged as a factor intensifying psychosocial stress, with unmarried women (i.e. single, divorced, or widowed) demonstrating elevated hair cortisol levels. The complexities of single parenting, the social stigma surrounding divorce, and the pervasive influence of a patriarchal society are plausible contributors to this observation [15]. Further, the cut flower industry provides infrastructure, schools, hospitals, and income for the residents of Naivasha [20], which likely contributes to our finding of lower stress (i.e. lower hair cortisol levels) in residents of Naivasha compared to Mogotio (**Figure 5**, Panel B). A different study did not observe any gender differences among Kenyan agricultural communities [12]. However, this finding may be influenced by a sampling bias. For instance, out of the 390 participants, only 28 were women (approximately 7% of the total participants) [12].

In addition to increased psychosocial stress, women had higher pesticide exposure than men. Women are crucial to the success of the cut flower industry in Naivasha; they hold only 12% of the jobs in Kenya but have 60% of the jobs in the cut flower farms [32,33]. The higher pesticide exposure raises concerns as maternal exposure to OPs and carbamates can produce adverse health outcomes, particularly neurotoxic damage in the developing foetus [34,35]. These concerns exist even where pesticide exposure occurs at levels where there is no apparent adverse health outcome for the mother, creating a ‘silent pandemic’ [36].

Gender-specific exposure reflects the complex socio-cultural dynamics in East Africa, where women play a pivotal role in the agricultural sector [34]. Regulations and formal training on pesticide use and associated health risks in the agriculture sector are not consistent and often less comprehensive than for those working on cut flower farms, elevating exposure risks to pesticide users. Recognition of the differences between the working and living conditions of men and women leads to a growing awareness of the importance of a gender-based analysis of human health and well-being in developing countries [34,35]. Gender equity must be a key indicator of a resilient, robust, sustainable, and secure agroecosystem. There is a need to develop new techniques for evaluating both occupational and non-occupational risks to women’s health. Indicators of women’s health and well-being must be measured and composited (i.e. a meta-indicator) to ensure an accurate assessment of their holistic health. If a validated meta-indicator suggests a decline, this should be taken as an early warning of pesticide exposure. This meta-indicator must be integrated into policy and practices to ensure the continuous improvement of women’s health and well-being, which is crucial for the survival and sustainability of the cut flower farms and the entire community.

### Limitations

There are several limitations to this study. First, using hair cortisol as a biomarker to assess perceived pesticide exposure was found to be ineffective. Hair cortisol levels are nonspecific biomarkers, serving as a cumulative integrator of stress [14]. Isolating the specific factor influencing hair cortisol levels (e.g. stress) among participants is challenging, given the diverse socio-cultural, economic pressures, and lifestyle factors impacting participants [14,15]. We also encountered non-response bias, as only 89 out of the 200 selected participants provided samples for the study. This bias led to a higher number of participants from Naivasha (n = 64) compared to Mogotio (n = 25) and a greater proportion of females (n = 59) than males (n = 30). This resulted in an unequal distribution across communities and genders, potentially affecting the representativeness of the study.

Since the completion of this project, cut flower farms have been established in Mogotio, starting from 2015 onwards. Future research should assess the impact of introducing flower farming to the region, specifically concerning risk attenuation. This assessment could provide additional insights into the potential benefits of the cut flower industry and its role in raising risk awareness within nearby communities. Future research should also ensure geographic and gender balance to ensure adequate representation.

## CONCLUSIONS

Cut flower industrial complexes increase risk awareness within local communities, leading to the adoption of risk management strategies and behavioural changes aimed at minimising exposure risks. Communities outside these industrial complexes exhibit increased rates of pesticide exposure, potentially due to a lack of awareness concerning pesticide risks. Risk awareness drives preventive action and is a key determinant of lowering exposure and vulnerability within communities. Despite exposure differences between communities, women are disproportionately affected, likely reflecting their socioeconomic status in African societies. African women serve as sentinels of ecotoxicological exposure, and monitoring women's health and well-being could serve as a preventive approach for identifying pesticide exposure in African communities.
